# Effects of diethylcarbamazine and ivermectin treatment on *Brugia malayi* gene expression in infected gerbils (*Meriones unguiculatus*)

**DOI:** 10.1017/pao.2019.1

**Published:** 2019-03-08

**Authors:** Mary J. Maclean, W. Walter Lorenz, Michael T. Dzimianski, Christopher Anna, Andrew R. Moorhead, Barbara J. Reaves, Adrian J. Wolstenholme

**Affiliations:** 1 Department of Infectious Diseases, College of Veterinary Medicine, University of Georgia, Athens, GA 30602, USA; 2 Center for Tropical and Emerging Global Diseases, University of Georgia, Athens, GA 30602, USA; 3 Institute for Bioinformatics, University of Georgia, Athens, GA 30602, USA; 4 Perthera Inc., McLean, VA 22102, USA

**Keywords:** Albendazole, *Brugia malayi*, diethylcarbamazine, ivermectin, transcriptome

## Abstract

Lymphatic filariasis (LF) threatens nearly 20% of the world’s population and has handicapped one-third of the 120 million people currently infected. Current control and elimination programs for LF rely on mass drug administration of albendazole plus diethylcarbamazine (DEC) or ivermectin. Only the mechanism of action of albendazole is well understood. To gain a better insight into antifilarial drug action *in vivo*, we treated gerbils harbouring patent *Brugia malayi* infections with 6 mg kg^−1^ DEC, 0.15 mg kg^−1^ ivermectin or 1 mg kg^−1^ albendazole. Treatments had no effect on the numbers of worms present in the peritoneal cavity of treated animals, so effects on gene expression were a direct result of the drug and not complicated by dying parasites. Adults and microfilariae were collected 1 and 7 days post-treatment and RNA isolated for transcriptomic analysis. The experiment was repeated three times. Ivermectin treatment produced the most differentially expressed genes (DEGs), 113. DEC treatment yielded 61 DEGs. Albendazole treatment resulted in little change in gene expression, with only 6 genes affected. In total, nearly 200 DEGs were identified with little overlap between treatment groups, suggesting that these drugs may interfere in different ways with processes important for parasite survival, development, and reproduction.

## Introduction

Human infections with filarial nematodes affect about 160 million people worldwide, with many more at risk of infection (Fenwick, 2012). Control of the major human filarial diseases, lymphatic filariasis (LF) caused by *Wuchereria bancrofti* and by *Brugia* spp, and river blindness caused by *Onchocerca volvulus*, is based on annual or semi-annual mass administration of anthelmintic drugs which remove microfilariae (Mf) from infected people for extended periods and thus interrupt transmission of the parasites *via* the insect vectors. The drugs used for LF are albendazole in combination with diethylcarbamazine (DEC), except in areas endemic for *O. volvulus* where it is used in combination with ivermectin. Ivermectin is currently the only anthelmintic used for control of river blindness (Gyapong *et al.,* 2005; Ottesen, 2006). Albendazole acts by binding to microtubules (Lacey, 1988) and hence blocking cell division and larval development (Cline *et al.,* 1992; Shenoy *et al.,* 1999), but the mechanisms of action of DEC and ivermectin against filarial parasites is less clear. Ivermectin irreversibly activates nematode glutamate-gated chloride channels (Cully *et al.,* 1994; Wolstenholme and Rogers, 2005), including those from filaria (Yates and Wolstenholme, 2004). The expression of these channels in the excretory/secretory pore of *Brugia malayi* Mf (Moreno *et al*., 2010) and reproductive tissue ofadult females (Li *et al*., 2014) correlates with the observed inhibition of secretion from Mf (Moreno *et al.,* 2010; Harischandra *et al.,* 2018) and the long-term sterilization of the females caused by the drug (Stolk *et al*., 2005). DEC has been reported to inhibit arachidonic acid metabolism in Mf (Maizels and Denham, 1992), blocking both cyclooxygenase and lipoxygenase.

Both DEC and ivermectin rapidly clear Mf from infected people (Hawking and Laurie, 1949; Basanez *et al*., 2008), an important part of their ability to block transmission of LF and onchocerciasis. However, *in vitro* assays do not accurately reproduce the *in vivo* potency of ivermectin against filarial parasites (Tompkins *et al.,* 2010; Storey *et al.,* 2014; Wolstenholme *et al*., 2016). For both DEC and ivermectin, it has been suggested that their activity against filarial parasites is aided by the host immune system (King *et al.,* 1982; Vickery *et al.,* 1985; Johnson *et al.,* 1988; Maizels and Denham, 1992; Ali *et al.,* 2002; McGarry *et al.,* 2005; Vatta *et al*., 2014; Kwarteng *et al*., 2016). Ivermectin specifically inhibits secretion from *B. malayi* Mf, which would include many immunomodulatory molecules produced by the parasites (Moreno and Geary, 2008; Moreno *et al.,* 2010). The only convenient small animal model for human filariasis is *B. malayi* infection in the Mongolian gerbil *(Meriones unguiculatus)* (Ash and Riley, 1970) yet even in this system albendazole, DEC and ivermectin have all been reported to be relatively ineffective (Denham *et al.,* 1978; Campbell, 1982; Gaur *et al.,* 2007). We are interested in the effects of the antifilarial drugs on the parasites *in vivo,* and considered that the absence of direct killing effect in the gerbil infection model could in fact be advantageous, since it would eliminate non-specific changes in gene expression that were due to parasite death and not a direct result of the drugs’ actions.

In order to gain further insights into the effects of the drugs programs we infected gerbils with *B. malayi* and, once the infection was patent, treated the animals with albendazole, DEC or ivermectin. Parasites were harvested 1 day and 1 week post-treatment and RNASeq used to probe changes in gene expression caused by the drugs. A similar approach has been used to examine changes in gene expression in adult female *B. malayi* caused by ivermectin and flubendazole exposure *in vitro* (Ballesteros *et al.,* 2016a; O’Neill *et al.,* 2016), and microarray experiments found changes in both host- and parasite-encoded genes following treatment of gerbils with DEC (Weinkopff *et al.,* 2008). The *in vivo* effects of doxycycline and tetracycline treatments on *Wolbachia* and *B. malayi* have been studied using microarrays (Ghedin *et al.,* 2009; Rao *et al.,* 2012) and Laing *et al.* (2012) examined the effects of ivermectin on a drug-resistant strain of *Caenorhabditis elegans,* but we believe that this is the first comprehensive attempt to study the effects of the anthelmintic drugs currently used in mass drug administration on gene expression, *in vivo*, for a human filarial parasite.

## Materials and methods

### Parasite infections of gerbils

*Brugia malayi* L3 larvae were provided by the Filariasis Research Reagent Resource (FR3) (Michalski *et al.,* 2011). All animal experiments were carried out in strict accordance with national and local guidelines and were approved by the University of Georgia IACUC (AUP A2015 08–039). Adult male gerbils *(Meriones unguiculatus)* were injected intraperitoneally with 300 *B. malayi* L3 and the infection allowed to become patent for 4 months. Gerbils were separated into one of four treatment groups: albendazole (Sigma, St Louis, MO, USA), DEC (DEC citrate salt, Sigma), ivermectin (Sigma) and control (1% v/v DMSO vehicle). Each gerbil was weighed to calculate the exact required dose prior to subcutaneous drug administration. The drug doses administered were: albendazole – 1 mg kg^−1^, DEC – 6 mg kg^−1^ and ivermectin – 0.15 mg kg^−1^. All the drug solutions were freshly made up immediately before administration to the infected animals. Twenty-four hours after treatment, half of the gerbils from each group (3) were euthanized by exposure to CO_2_ for parasite collection. The other half of the gerbils were euthanized 7 days after drug treatment. This experiment was repeated in triplicate. After euthanasia the peritoneal cavity was opened with a scalpel to allow collection of adult parasites with tweezers. Mf were collected *via* peritoneal lavage with Earle’s Balanced Salt Solution (EBSS) (Life Technologies, Carlsbad, CA, USA). After collecting worms from the peritoneal cavity, the abdominal cavity and testes were also examined for additional adult parasites. Upon retrieval, adult parasites were placed in 90 mm petri dishes in EBSS before being counted by sex and washed twice more. Mf were placed in 50 mL centrifuge tubes and the tubes were filled to capacity before Mf were counted. They were then centrifuged at 1200 × ***g*** for 10 min. This process was carried out twice more for three washes. Half of the Mf from each treatment group were exsheathed with calcium chloride. 20 mm of calcium chloride was added to a tube containing Mf in EBSS, shaken, and incubated at 37 °C for 30 min as described (Devaney and Howells, 1979). After the final washing, 10–15 adults (by sex, treatment group) and ~250 000 Mf were placed in a 1.5 mL microcentrifuge tube with 1 mL of RNAlater® (Sigma-Aldrich, St. Louis, MO) and incubated at 4 °C overnight before being transferred to −80 °C, per manufacturer’s instruction.

### RNA extraction

All surfaces (including gloves) and pipettes used for RNA extraction were treated with RNase AWAY™ (Life Technologies, Grand Island, NY, USA). Tubes were treated with diethylpyrocarbonate (DEPC; Sigma) to inhibit RNase. All water used in the process was nuclease-free. RNA was extracted from frozen *B. malayi* adults and Mf. Sample preparation for adults was carried out with several similar, but slightly varied methods. The majority of samples were obtained using the RNeasy Plant Mini Kit (Qiagen, Germantown, MD, USA). Lysis buffer RLT with 10 *μ*L 2-mercaptoethanol/mL^−1^ was added to the frozen sample for homogenization with a mortar and pestle. Liquid nitrogen was added to the sample to re-freeze it and aid in tissue disruption. After crushing with liquid nitrogen twice, the sample was aliquoted into DEPC-treated tubes to which additional lysis buffer was added. A 25-gauge needle (Becton Dickinson, Franklin Lakes, NJ, USA) was used to further homogenize tissue, which was drawn up and expelled from the syringe 25 times. Lysed tissue was transferred into a QIAshredder spin column, and RNA was cleaned and eluted following kit instructions. Samples were also obtained using the Direct-zol™ RNA Mini Prep Kit (Zymo Research, Irvine, CA, USA). Samples were homogenized as above using TRIzol® Reagent (Life Technologies) and aliquoted into multiple tubes before being centrifuged for 10 min at 12 000 × ***g*** to pellet the solid material. The supernatant was transferred to a new tube and RNA was purified and eluted following the kit protocol. The ‘LogSpin’ method developed by Yaffe *et al.* (2012) was also used, combining homogenization in TRIzol® and purification and washing with mini prep columns and sodium acetate and 75% ethanol.

RNA extraction from Mf was mainly performed using a modified TRIzol® protocol. 1.5 mL of TRIzol® was added to one sample of ~500 000 frozen *B. malayi* Mf and homogenized using a mortar and pestle, with the sample repeatedly frozen and crushed with liquid nitrogen. Samples were aliquoted three times into 2 mL tubes with additional TRIzol® added. A 25-gauge needle, followed by a 30-gauge needle was used to further homogenize tissue, which was drawn up and expelled from the syringe 25 times. Tubes were centrifuged for 10 min at 12 000 × ***g*** to pellet solid material. The supernatant was added to a new tube and mixed with 0.2 mL of chloroform (Sigma), and shaken for 30 seconds. After a 3-min incubation at room temperature, the samples were centrifuged again for 15 min under the same specifications. The aqueous layer was added to an RNA Clean and Concentrator™ spin column (Zymo Research) and the procedure was completed as per product instructions. RNA samples eluted in nuclease-free water were analysed on a NanoDrop 2000 Spectrophotometer (Thermo Fisher Scientific, Pittsburgh, PA) to determine their concentration and to measure the 260/280 absorbance ratio. Samples with a 260/280 ratio above 1.75 were analysed for integrity on an Agilent 2100 Bioanalyzer (Agilent Technologies, Santa Clara, CA, USA) using an RNA 6000 Nano chip.

### RNASeq analysis

Total RNA (1–100 *μ*g), was isolated from groups of pooled individuals for each treatment and time point. The number of individuals required per sample to yield sufficient RNA ranged from female, 8–25; male, 25–60; and Mf, 2.5–5.0 × 10^5^. RIN values ≥7.0 were obtained for adult worm samples while values for Mf samples were typically lower, with a minimum RIN score ≥5.5. Three biological replicates were performed and analysed for all samples except for the Mf 7 days post-treatment, where RNA for only two replicates was submitted for sequencing. Samples were submitted to the UGA Georgia Genomics Facility and libraries were synthesized using a TruSeq Stranded Total RNA LT Sample Prep Kit (Illumina, San Diego, CA, USA). Paired-end sequencing was performed on three Illumina NexSeq 500 runs (150 bp, high output).

Raw read data were demultiplexed and converted to fastqc using bcl2fastq from Illumina. Bioinformatics analyses were carried out by the UGA Quantitative Biology Consulting Group as follows: Raw and trimmed read data were quality assessed using FastQC (ver. 0.11.4) software (https://www.bioinformatics.babraham.ac.uk/projects/fastqc/) and residual adapter/index removal and quality trimming was done with Trimmomatic software (Bolger *et al.,* 2014). A minimum read length cutoff of 50 bases was employed and the average sample quality was typically above QC 30. The average loss of data after trimming was typically between 5 and 10%. Only surviving paired reads were used in subsequent alignments to reference genomes. The *B. malayi* genome (build WS253) and GFF3 file were downloaded from Wormbase (ftp://ftp.wormbase.org/pub/wormbase/releases/WS253/species/b_malayi/PRJNA10729/) and the *Wolbachia* endosymbiont (strain TRS) reference genome from NCBI (at ftp://ftp.ncbi.nlm.nih.gov/genomes/archive/old_refseq/Bacteria/Wolbachia_endosymbiont_TRS_of_Brugia_malayi_uid58107/), respectively. Bowtie2, ver. 2.2.3, (Langmead and Salzberg, 2012) indices were constructed for both. The *B. malayi* GFF3 file was converted to GTF format using the gffread script Cufflinks, ver. 2.2.1, (Trapnell *et al.,* 2010) followed by file sorting with Integrated Genomics Viewer software (Robinson *et al.,* 2011). Each sample was mapped to the *B. malayi* genome using Tophat2, ver. 2.0.14, (Kim *et al.,* 2013) run at default settings. Mapping resulted in a typical concordant pair alignment of >90%. Several libraries were also aligned to the *Wolbachia* genome using Tophat2 and, in those cases, the concordant pair alignment rate was ≤0.1% for all samples tested indicating that filtering for *Wolbachia* contamination was unnecessary. Custom Perl scripts were used to: (1) generate run scripts for name sorting each Tophat BAM file with SAMtools (Li *et al.,* 2009) and (2) generate scripts for count extraction using HTSeq (Anders *et al.,* 2015). HTSeq output files containing gene-to-count information for each sample were concatenated with AWK to generate the final count matrices used in subsequent expression analyses. The DESeq2 Bioconductor package (Love *et al.,* 2014) was used in the R computing environment to normalize count data, estimate dispersion and fit a negative binomial model for analysis of differentially expressed genes (DEGs). For each comparison group, a metadata table was generated containing the relevant sample and replicate associations and a secondary (batch) analysis column was included to account for the differences in replicate sample collection times within each group. *P* values were adjusted for multiple testing for determination of false discovery rate (FDR) using the Benjamini-Hochberg correction (Benjamini and Hochberg, 1995). Regularized logarithm (rlog) transformation of raw count matrices was used for principal component analysis (PCA).

Differential expression results were filtered based on a FDR of less than 5% and output was imported into Microsoft Excel for the inclusion of gene annotations and sorting. DEGs were designated by their WormBase Gene ID numbers (ex. WBGene00223871), and were translated into their respective *B. malayi* gene names (ex. Bm3610) using WormBait. This program was designed specifically for this study and is freely available for download at https://github.com/c-anna/WormBait. This program uses the WormBase public RESTful API (http://www.wormbase.org/about/userguide/for_developers/API-REST#10-10) to collect various pieces of information about genes and proteins such as orthologs, gene classes, and gene models, quickly compiling information and producing a CSV report with the collected information suitable for further review in Microsoft Excel.

Sequence data are available at NCBI BioProject ID PRJNA388112.

### Quantitative polymerase chain reaction (qPCR)

Sequences from WormBase GeneIDs were used to design primers for selected DEG hits using the GenScript Real-time PCR (TaqMan) primer design tool (www.genescript.com/ssl-bin/app/primer). Design parameters were set to return primers and probes for amplicons between 50 and 150 bp and the same ranges for minimum and maximum melting temperatures. Each primer set was designed to span an exon-exon junction on either side, preventing any contaminating gDNA in each sample from being amplified. Two endogenous housekeeping genes, NADH dehydrogenase subunit 1 (NADH), and histone H3 were chosen as internal controls. The primer/probe sets for NADH1 and histone H3 were chosen based on their use as endogenous controls in earlier studies (Li *et al.,* 2004; Lustigman *et al.,* 2014). The primer set for NADH1 was not explicitly designed to span exons because it could not be manually defined due to discrepancies in gene naming conventions in WormBase and NCBI. The histone H3 probe was explicitly designed to span exons, based on the availability of the sequence in WormBase. Primer/probe sets for adults were designed based on DEGs compiled from the WS249 release of WormBase. With the release of the WS253 assembly, adult gene hits were compiled again alongside Mf gene hits. Details of the primers and probes used are in Supplementary Table 1. Primer and probe sets for the target gene of interest and the histone H3 endogenous control were prepared in quadruplicate. Each reaction was run on an Mx3000P Thermocycler (Stratagene) using Mx3000 software v 4.01. Fluorescence threshold data (Ct values) were collected for ROX (reference dye), HEX (histone probe), and FAM (target probe). Reactions were run for 40 cycles with an annealing temperature of 55 °C for 1 min and an extension temperature of 60 °C for 1 min.

## Results

### Drug treatment of infected gerbils

None of the drugs we tested have been reported to have any phenotypic effect on Mf *in vitro* at concentrations relevant to those observed in treated people. Since it has been suggested that the activity of DEC and ivermectin might depend on the host immune system, we decided to study the effects of *in vivo* administration of the anthelmintic drugs on gene expression in adult and Mf stages of *B. malayi.* As the gerbil is the only convenient small animal model for *B. malayi* infection, we used gerbils that had been infected with about 300 intraperitoneal L3; this infection route was chosen because of the greater ease of recovery of the parasites after treatment. We treated animals harbouring a patent infection, 4 months post-inoculation of the L3, with either albendazole, DEC or ivermectin at concentrations equivalent to those used in the LF elimination programs. Parasites were harvested from the peritoneal cavities of euthanized gerbils 24 h or 7 days posttreatment. None of the drug treatments had any significant effect on the number of adult or larval worms recovered at these times (Table 1).

### Transcriptomic effects of the drug treatments on adult and larval B. malayi

RNA was isolated from all of these parasite populations and subjected to Illumina sequencing. A total of 1.1 × 10^8^ total paired reads were obtained, resulting in a collective 3102 × coverage of the *B. malayi* transcriptome and 1898 X coverage of the *B. malayi* genome (Ghedin *et al.,* 2007). Eighty percent or more of reads from each of the three sequencing runs mapped to the reference genome concordantly. The average overall mapping rate was 91.8% for adults and 82.9% for Mf. Reads covered 98% or more of the WS253 release of the *B. malayi* genome assembly (Supplementary Table 2).

**Fig. 1.**
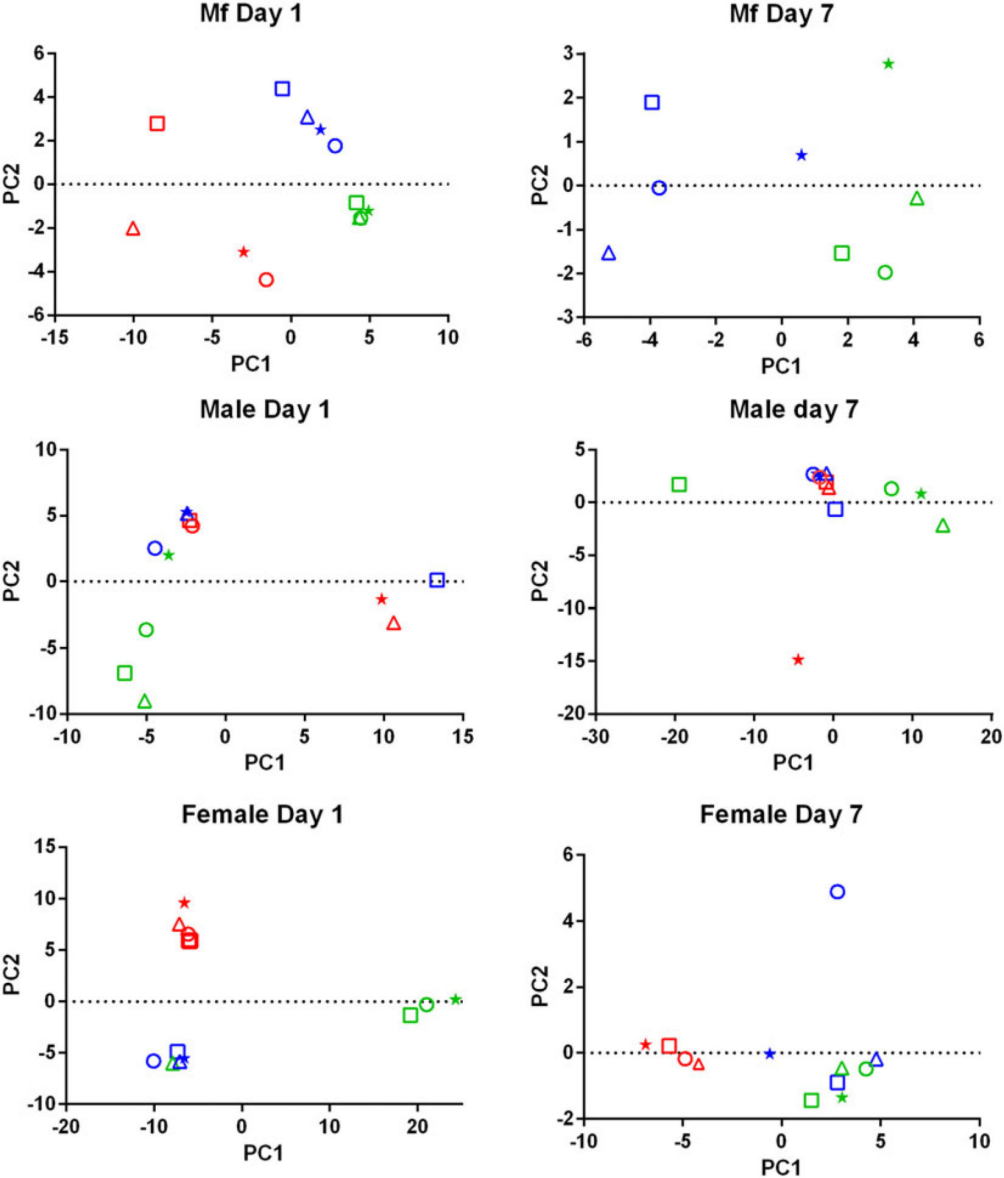
Principal Component Analysis of the RNASeq data. For each set of samples (Mf, adult males and adult females at 1 day and 7 days post-treatment) the replicates are colour-coded; replicate 1 is in red, replicate 2 is in blue and replicate 3 is in green. For each replicate the principal components are shown for the control samples (o), albendazole-treated (Δ), DEC-treated (*) and ivermectin-treated worms (□).

PCA of the transcriptomic data (Fig. 1) showed that the data from parasites collected from different treatment groups within an individual experiment tended to be more similar to each other than to those of the same treatment groups in the three replicates. Despite the use of an inbred laboratory strain of *B. malayi* and host animals purchased from the same vendor over a fairly short period of time, the batch effects between experimental groups were more pronounced than the effects of treatment.

**Fig. 2.**
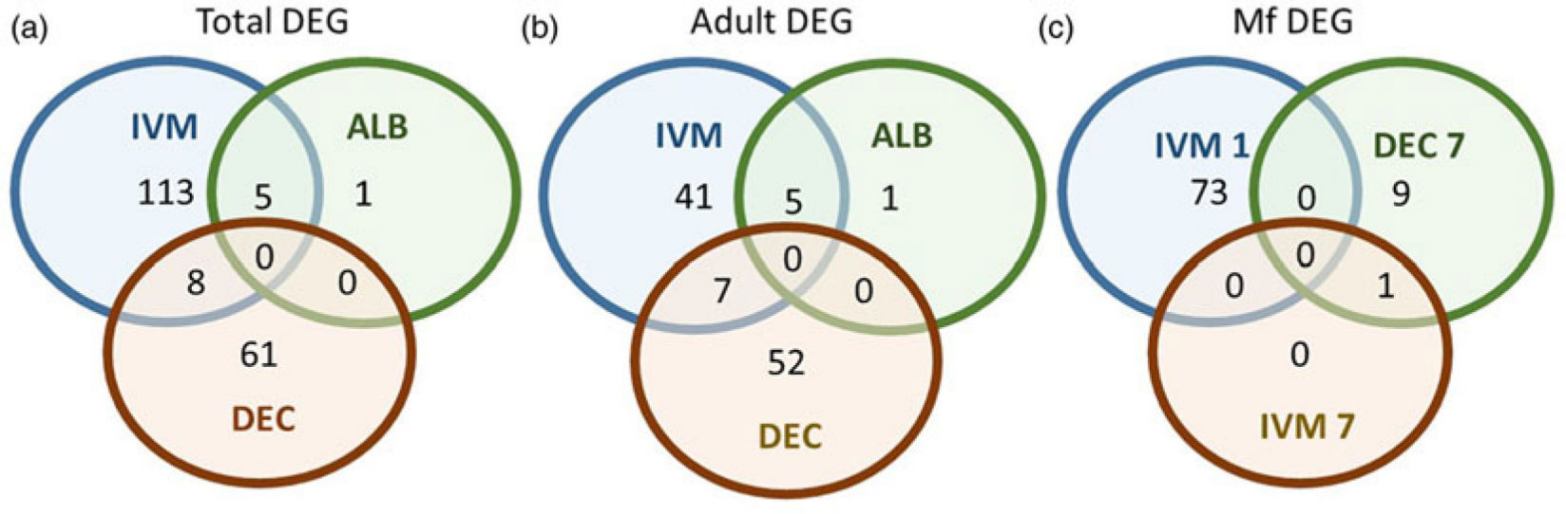
Limited overlap in differential gene expression between drug treatments. (a) Of the 203 differentially expressed genes, only 13 were affected by more than one drug treatment at any life-stage. Five genes were differentially expressed in both ivermectin- and albendazole-treated worms and eight in both ivermectin- and DEC-treated worms. (b) Of the 119 genes whose expression was affected in adult worms, five were altered in both ivermectin- and albendazole-treated parasites and seven in ivermectin- and DEC-treated ones. (c) Of the 83 genes whose expression was altered in Mf at any time following treatment with any drug, only one was affected following either ivermectin or DEC, at 7 days post-treatment. IVM 1 is the group of DEG 1 day after treatment with ivermectin, IVM 7 is the DEG group after 7 days and DEC 7 is the group of DEG 7 days after treatment with DEC.

A total of 119 DEGs, that is genes whose expression differed in a statistically significant way between drug-treated and control samples, were identified in adult males and females, and 84 were found in Mf (Table 2; Supplementary Table 3). DEGs were grouped by drug treatment (Fig. 2a, and life stage (Fig. 2b and c)) to visualize their number and distribution. Of the 203 identified DEGs, 189 were specific to a particular drug treatment and/or parasite life stage. There were few overlaps in the DEGs among any of the treatment groups, or between males, females and Mf. Five of the 6 DEGs seen in the albendazole-treated female worms were also affected by the ivermectin treatment of female worms, with very similar levels of change observed. The only gene uniquely affected by the albendazole treatment was Bm8178, an ortholog of the *C. elegans* K05F1.9 and ZK354.7 genes. In *C. elegans*, these genes are affected by various noxious stimuli and are enriched in the germ line (Ortiz *et al.,* 2014). No DEGs were observed in males or Mf following albendazole treatment. There were eight genes whose expression was significantly altered by both DEC and ivermectin, seven in females and one in Mf, at 7 days post-treatment.

Gene annotation information for DEGIDs were parsed from WormBase (www.wormbase.org) using the program WormBait (https://github.com/c-anna/WormBait) and included orthologs identified in *C. elegans*, *O. volvulus* and *Homo sapiens*. Gene ontology (GO) and pathway analysis was carried out with PANTHER (Mi *et al.,* 2016) using the *C. elegans* orthologs of *B. malayi* gene hits (Supplementary Table 4).

### DEGs following DEC treatment

One day after treatment with DEC only five DEGs were identified, all in male worms; in contrast, there were no DEGs in males 7 days after treatment, whereas there were multiple genes showing changes in expression in both females and Mf at this time point (Table 2). The ortholog of *cdh-1,* Bm8476, encoding a cadherin, was the only gene upregulated by DEC treatment of males; the other male-specific genes were downregulated and have functions associated with metabolic processes. Seven days post-treatment, most of the DEGs in females were upregulated and five biological processes including RNA metabolism and regulation of RNA pol II transcription, as well as pathways related to Alzheimer Disease-presenilin and Wnt signaling, were significantly overrepresented in this group (Table 3). All of the female downregulated DEGs at 7 days post-treatment were annotated with ‘cellular process’ as the only biological process, with binding and catalytic activity as the molecular function. In Mf, nine of the 10 DEGs were downregulated 7 days post-treatment. No GO terms were significantly over-represented in this group. The only upregulated transcript, Bm1750, is an ortholog of *hlh-1,* a helix-loop-helix transcription factor.

### DEGs following ivermectin treatment

In ivermectin-treated worms, some genes were differentially expressed 1 day after treatment in males and Mf, but not females (Table 2). The Mf produced the largest number of DEGs for any group 1 day after treatment with ivermectin. The number of DEGs in males seen at 1 or 7 days post-treatment was similar, although there was not a single gene whose expression was altered at both times. Seven days after treatment only one gene, Bm41, was differentially expressed in Mf, and expression of this gene was also changed in DEC-treated Mf at this time. Expression of Bm41 decreases during L2 development in the mosquito vector (Choi *et al*., 2014). Twenty-five transcripts were significantly affected in females 7 days post-treatment. Collectively examining transcripts in adults and Mf that were up- or downregulated by ivermectin, six biological processes were shared including cellular component organization or biogenesis, cellular process, developmental process, localization, metabolic process and multicellular organismal process. Six additional processes were only associated with upregulated genes: biological adhesion, biological regulation, immune system process, locomotion, reproduction and response to stimulus (Table 3). Across all transcripts upregulated by ivermectin, products of genes involved in the endothelin signaling pathway were overrepresented in ivermectin-treated Mf 1 day post-treatment, including Bma-AEX-5, Bma-GCY-9 and Bma-LIT-1. In the Mf, there was also significant over-representation of the biological processes chromosome segregation, metabolic process and tubulin complex in the upregulated transcripts, suggesting that cell division might be affected by the drug treatment. There were no significantly over-represented GO terms in the downregulated genes, all of which were involved in cellular or metabolic processes. In males, all DEGs at 1 day were downregulated, with cytokinesis and motor activity significantly over-represented GO terms in this group. Nicotinic acetylcholine receptor signalling was the only over-represented pathway. At 7 days, three of the up-regulated transcripts in males, Bm7894, Bm9729 and Bm8043, encoded collagens. In females the only GO term over-represented amongst the DEGs at 7 days was a developmental process. One gene, Bm17065, was downregulated in females 7 days post-treatment, but upregulated in males at the same time-point. Bm17065 encodes a small protein with no annotated functions or C. *elegans* ortholog. Seven genes, Bm5144, Bm6215, Bm6122, Bm6123, Bm3958, Bm1811 and Bm5103, were upregulated in females 7 days post-treatment with both DEC and ivermectin. These genes encode HIL-1 (DNA-binding protein), LIT-1 (protein phosphorylation), two calcium-binding cell adhesion proteins, SOX-2 (transcription factor), LAT-1 (G-protein coupled receptor) and HLH-2 (transcription factor), respectively.

### qPCR confirmation of expression changes

In order to confirm the changes in gene expression identified by RNASeq, we carried out qPCR on selected target genes to compare expression between single treated samples and time-matched controls. The target genes (Supplementary Table 4) were chosen to represent both time points and all treatments in males, females, and Mf, and included both up- and downregulated genes. For all nine target genes tested, changes in gene expression from qPCR were in the same direction as those given by DESeq2, though in several instances the qPCR data suggested that the magnitude of the changes was greater than the DESeq analysis.

## Discussion

Drug treatment of the *B. malayi* infection in gerbils had no effect on the parasite numbers in the peritoneal cavity either 1 day or 7 days after treatment (Table 1). Though this may seem to be initially surprising it is consistent with previous observations. Treatment of sub-cutaneous *B. pahangi* infections of gerbils with an oral dose of 0.2 mg kg^−1^ ivermectin had no effect on adult worm numbers, did not clear circulating Mf, and only reduced their numbers by ~85% 3 weeks after treatment (Rao *et al.,* 1990; Bosshardt *et al.,* 1995). Treatment of a peritoneal *B. malayi* infection with 0.2 mg kg^−1^ ivermectin also had no effect on adult worm numbers 1 week after treatment (Dooley *et al*., 2015). For DEC, 5 days of treatment with 100 mg kg^−1^ (more than 10 times the dose used here) did cause a transient clearance of *B. malayi* Mf and a 50% reduction in adult worms. The same treatment regime with albendazole (100 times the treatment used here) had no effect on Mf numbers until 21 days post-treatment, but did clear adult worms (Rao *et al*., 2008). The absence of any measurable parasite killing in these experiments had one clear benefit; it allowed us to examine the effects of exposure to the drugs *in vivo* on the parasite transcriptome in the absence of any non-specific changes in gene expression due to dead or dying parasites. This approach was vindicated by the clear differences between the results obtained following treatment with the three drugs, which we believe are due to the direct effects of the drugs on the nematodes. The absence of any genes that were affected by all of the treatments is probably due to the absence of non-specific changes associated with parasite death. Overall, we observed changes in the expression of a small number of genes. Both the number of DEGs and the fold changes in expression that we observed following treatment were smaller than we predicted. Part of the explanation for this was batch effects revealed by the PCA, showing that gene expression in parasites of different treatment groups in the same experiment tended to be more similar than that in parasites of the same treatment group in different experiments (Fig. 1). This was despite the inbred nature of the *B. malayi* strain used (Michalski *et al*., 2011), the common source for the gerbils and our best attempts to maintain a consistent environment between the three biological replicates; this observation emphasizes the variability in gene expression likely to be observed between infections even with inbred parasite and host populations. This level of variation between the biological replicates may also explain the differences in the apparent extent of the differential expression we saw between the RNASeq analysis and the qPCR data (Supplementary Table 4), as the latter were obtained from only one of the experiments used in the former.

There were clear differences in the results obtained following treatment with the three drugs, with albendazole treatment resulting in changes in the expression of only a few genes, all in females 7 days post-treatment. Albendazole acts *via* inhibiting the polymerization of nematode tubulin (Lacey, 1988), though an effect on the *Wolbachia* endosymbiont has also been postulated (Serbus *et al*., 2012). The effectiveness of the albendazole treatment may have been reduced by the need for its metabolic activation causing a reduced bioavailability (Marriner and Bogan, 1980; Hennessy *et al*., 1989). These results will not be considered further, except to note that this treatment group acts as a further control for non-specific variations in transcript levels and strengthens the case that the changes in gene expression seen after DEC and ivermectin treatment were drug-specific and a direct consequence of treatment with those compounds. This outcome was different from that reported after *in vitro* exposure of *B. malayi* female worms to flubendazole (O’Neill *et al.,* 2016) but for the reasons given above it seems unlikely that the parasites in our experiment were exposed to drug concentrations at all close to those used in the *in vitro* experiment.

The DEC and ivermectin treatments produced marked gender-specific effects on gene expression in adult worms. Effects on gene expression in males became apparent earlier than in females after both drugs, though ivermectin had a more pronounced effect on Mf than did DEC. The faster effects on males may be due to their smaller size and more rapid penetration of the drugs. The Mf data suggest that ivermectin does more direct damage to them than does DEC, as 24 h post-treatment ivermectin-treated Mf had the highest number of DEG of any of the experimental groups – 73. This result suggests that the drug treatment is having a rapid effect on the Mf, even though this does not result in any parasite death. In the context of a human infection, it may be that similar changes have a sufficiently deleterious effect on Mf to lead to their rapid removal from the circulation. After DEC treatment, changes were seen at 24 h in the expression of a handful of genes in adult males, with effects on females and Mf only observed 7 days after treatment. The limited overlap in the transcripts affected by DEC *vs* ivermectin treatment suggests that the effects we saw were specific to the two drugs and did not reflect generalized responses to drug-induced damage. The seven genes upregulated in female worms treated with either DEC or ivermectin do indicate some common responses, with increases in some transcription factors and cell-adhesion molecules. None of the genes whose expression was altered in the DEC- and ivermectin-treated animals was directly related to the presumed drug targets, arachidonic acid metabolism or glutamate-gated chloride channels, respectively (Maizels and Denham, 1992; Cully *et al.,* 1994; Wolstenholme and Rogers, 2005; Wolstenholme, 2011). Ivermectin treatment was associated with a reduction in transcripts involved in motor activity and nicotinic receptor signalling in males after 24 h, which could argue for an effect of the drug on locomotion. *In vitro,* males are paralyzed more rapidly than females by exposure to ivermectin (Tompkins *et al.,* 2010), so they might be more susceptible to this effect of the drug. Alternatively, given that this effect was not seen in males after 7 days, it may be that a similar transient effect also took place in females but between 1 and 7 days post-treatment and so was not detected by our experimental design. Ivermectin and DEC both cause a long-term reduction in Mf numbers in infected people (Stolk *et al*., 2005), which has been correlated with expression of the glutamate-gated chloride channels in the reproductive tissues (Li *et al.,* 2014). In this context, it is interesting that ‘developmental process’ was the only GO term overrepresented amongst the genes whose expression was increased 7 days post-ivermectin expression, which might reflect the inhibition of Mf development and release caused by the drug. These genes included the orthologs of the *C. elegans* genes *hil-1, lit-1, hmr-1, sox-2, lat-1* & *hlh-2* (Kaletta *et al.,* 1997; Costa *et al.,* 1998; Portman and Emmons, 2000; Jedrusik *et al.,* 2002; Langenhan *et al.,* 2009; Kagias *et al.,* 2012). In contrast, only one of the genes whose expression was decreased by the drug treatment, Bm2841, has a *C. elegans* ortholog, *oac-9.*

Taken together, the picture that emerges is of early effects of ivermectin on Mf, consistent with a direct action of the drug against the larvae, which may then facilitate clearance from the circulation in human hosts, possibly involving an immune response against a weakened parasite population. In addition to increased transcription and possibly cell division in the Mf, the identification of increased endothelin-like signalling may point to heightened production of specific signalling peptides. In *C. elegans* GCY-9 is responsible for carbon dioxide avoidance *via* increased expression of FLP-19 (Smith *et al.,* 2013; Romanos *et al.,* 2017); while *B. malayi* Mf may not normally be exposed to higher concentrations of carbon dioxide, this upregulation may reflect their detection of and response to deleterious effects caused by the drug. Bm6544 is one of a group of differentially-expressed genes whose *C. elegans* ortholog expression is regulated by noxious stimuli or might otherwise be predicted to respond to stress (Supplementary Table 5); a single ABC transporter transcript (Bm3156, which encodes an ortholog of *C. elegans wht-4,* an ABCG sub-family member) was upregulated in Mf 24 h post-treatment. ABC transporters have been proposed to be the major mediators of ivermectin detoxification by nematodes (Lespine *et al.,* 2012) and ABCG transporters are involved in a wide variety of transport processes, including lipophilic drugs (Velamakanni *et al.,* 2007).

The previous transcriptomic studies of drug treatment on *B. malayi* was carried out *in vitro* using adult females and used different mapping platforms and parameters for identifying DEGs (Ballesteros *et al.,* 2016a; O’Neill *et al.,* 2016). Our *in vivo* study should have removed the complicating effects of *in vitro* culture on these parasites (Ballesteros *et al.,* 2016b). We found no changes in the expression of any genes in adult female worms 24 h post-treatment with any of the drugs, compared with the 34 DEG found by Ballesteros *et al.* (2016*a*) following *in vitro* exposure to 100 nm ivermectin. One immediate difficulty in comparing the two sets of data is that we do not know what effective concentration of ivermectin the parasites were actually exposed to in our *in vivo* experiment and little or nothing is known about the pharmacokinetics of the drug in gerbils. In mice, a sub-cutaneous injection of 0.2 mg kg^−1^ ivermectin resulted in a plasma concentration of ~100 nM 2 h post-injection, falling by about 60% at 24 h (Kiki-Mvouaka *et al.,* 2010). If those concentrations are also maintained in the peritoneal cavity and the pharmacokinetics of the drug in gerbils is similar to that in mice, then the parasites in both *in vivo* and *in vitro* experiments might have been exposed to similar drug concentrations. It seems unlikely that the 300 nm and 1 *μ*m ivermectin treatments carried out by Ballesteros *et al.* (2016*a*) reflect drug exposure in our infected animals. However, they were some common DEG between the two studies. Three of our hits, Bm10580, Bm6544 and Bm8043 are regulated *in vitro* by ivermectin (Ballesteros *et al.,* 2016a). Bm4605, Bm8043, Bm7894 and Bm9021 are four cuticle collagens whose expression was up-regulated in males 7 days post-treatment with IVM *in vivo*, whereas expression of these same genes was downregulated in females treated *in vitro* with IVM for 5 days (Ballesteros *et al.,* 2016a); these genes were up-regulated *in vitro* in female nematodes that were not treated with ivermectin (Ballesteros *et al.,* 2016b). These changes in collagen expression may reflect damage and repair to the cuticle following drug exposure or *in vitro* cultivation.

In summary, we found few changes in gene expression that were due to albendazole treatment. For DEC, there are early limited effects on males and more delayed and extensive effects on gene expression in females and in Mf. Ivermectin caused a rapid change in gene expression in Mf, from which they largely recovered by 7 days post-treatment, whereas effects on female gene expression were only apparent after 7 days. Male gene expression was altered at both time points, though different genes were affected 1 day and 7 days after treatment. The early effect of ivermectin on males seemed to be transient and muscle function was a major target. Though this study does not provide a definitive mechanism for the antifilarial effect of DEC or ivermectin, it does confirm the complexity of those effects and suggest future avenues for investigation, such as using RNAi to knock-down the transcripts identified here. Current and future studies are aimed at confirming the role of the DEG identified here in the mode of action of these drugs.

## Supplementary Material

Supplementary Table 1

Supplementary Table 2

Supplementary Table 3

Supplementary Table 4

Supplementary Table 5

## Figures and Tables

**Table 1.** Parasite recovery following drug treatment

	Control			ALB			DEC			IVM		
	♂	♀	Mf^a^	♂	♀	Mf^a^	♂	♀	Mf^a^	♂	♀	Mf^a^
Round 1: 24 h	46	84	1.5	104	120	3.5	32	56	1.2	62	109	2.3
Round 1: 1 week	115	112	7.6	62	56	4.8	81	115	6.4	118	114	5
Round 2: 24 h	148	175	7.0	166	155	6.8	168	132	5	48	39	2.2
Round 2: 1 week	78	72	3.2	99	95	4.3	179	191	4.6	112	104	3
Round 3: 24 h	79	43	2	86	49	2.9	70	58	2.7	89	128	3.4
Round 3: 1 week	146	100	3.9	115	93	3.8	91	102	4	207	157	4.4
Gender total	612	586		632	568		621	654		636	651	
Experiment total	1198		25.2	1200		26.1	1275		23.9	1287		20.3

a Mf counted in millions.

**Table 2.** Summary of Differentially Expressed Genes (DEG)

Drug	ALB						DEC						IVM					
	24 h			7 days			24 h			7 days			24 h			7 days		
	Mf	♀	♂	Mf	♀	♂	Mf	♀	♂	Mf	♀	♂	Mf	♀	♂	Mf	♀	♂
Total DEG	0	0	0	0	6	0	0	0	5	10	54	0	73	0	14	1	25	15
Downregulated DEG	0	0	0	0	6	0	0	0	4	9	5	0	12	0	14	1	12	3
Upregulated DEG	0	0	0	0	0	0	0	0	1	1	49	0	61	0	0	0	13	12
DEG with *C. elegans* ortholog	0	0	0	0	2	0	0	0	5	5	42	0	39	0	11	0	14	8
DEG with GO terms	0	0	0	0	1	0	0	0	3	5	42	0	36	0	6	0	15	7

**Table 3.** GO Terms over-represented within the Differentially Expressed Genes (DEG)

		1 Day post-treatment	7 Days post-treatment
Albendazole		None	None
DEC	Mf	None	None
	Male	None	None
	Female	None	RNA metabolism Regulation of RNA Pol II transcription DNA-dependent transcription Chromatin, chromatin-binding Alzheimer-disease/presenilin signaling Wnt signalling
Ivermectin	Mf	Chromosome segregation Metabolic process Tubulin Complex Endothelin signalling	None
	Male	Cytokinesis Motor activity Nicotinic Acetylcholine Receptor signalling	None
	Female	None	Developmental Process
