# Supplementary material for: Effects of diethylcarbamazine and ivermectin treatment on Brugia malayi gene expression in infected gerbils (Meriones unguiculatus)
Source: Parasitol Open. Author manuscript; Available in PMC 2021 Mar 26. (PMC7994942; doi:10.1017/pao.2019.1)
Supplement: Supplementary Table 1 [file NIHMS1519550-supplement-Supplementary_Table_1.docx]

*Supplementary Table 1*. qPCR primers and probes

| GeneID/  Symbol | Primer  Set | Sequence (5’ to 3’) | Product size |
| --- | --- | --- | --- |
| Bm7847 | F | AACGACAACCTCGTCAAACA | 69 bp |
|  | R | TCCGCCTAATTTAACGTCATC |  |
|  | Probe | (FAM)-CACGCATGAGCCATTCAATCGA-(BHQ) |  |
| Bm6220 | F | TGATGTTGACTGTCGCAGAA | 142 bp |
|  | R | AGTTTCACCGTCGATCCTTC |  |
|  | Probe | (FAM)-CCGTGTACCGGGTACATCAGTCGA-(BHQ) |  |
| Bm1750 | F | ACAGGATGCTAAAGAACATGCAGTAGA | 108 bp |
|  | R | GCGACATCCGCATTTGTCTGA |  |
|  | Probe | (FAM)-TCGCTGGTTGAACCACCTGCTGCCGA-(BHQ) |  |
| Bm4783 | F | ACCAACAGGGCCTACTAACATCAC | 79 bp |
|  | R | GGCAACGTAGCTGCGATAGG |  |
|  | Probe | (FAM)-CGGTTAGCAACTTCCGCCAGCTGCT  GC-(BHQ) |  |
| Bm4605 | F | CCAGATGAACCACTTCAACG | 62 bp |
|  | R | GGTCCTTGTGGTCCTTGTTC |  |
|  | Probe | (FAM)-TCCTGGTTCGCCCGGAGC-(BHQ) |  |
| Bm3390 | F | GTTAATGCGTAGCCGAGACC | 88 bp |
|  | R | ACGTTGTAACGCTGCTTGTC |  |
|  | Probe | (FAM)-CCCGGAATACCCTGAAGATTCCG-(BHQ) |  |
| Bm5185 | F | ATTCAAATCATCCAATCCCAA | 78 bp |
|  | R | TGCCACCTTTACTACCTCCA |  |
|  | Probe | (FAM)-CACCGCGCACTGATGGTGAC-(BHQ) |  |
| Bm4155 | F | CGTCGGTTAACACGCAAAGGTAAA | 108 bp |
|  | R | AACTTCTGCCTCAGATTGACTTGC |  |
|  | Probe | (FAM)-ACGTCGTCGCTCAACGGCTACCACG  G-(BHQ) |  |
| Bm4360 | F | TTCAAGCGAGAAGGTGCGGAT | 127 bp |
|  | R | TGATGTCGTTGATACGCATCGGA |  |
|  | Probe | (FAM)-ACAAGCTGGCGCTACGCGACGCT-(BHQ) |  |
| Bm1_50575  NADH | F | TGGTACAAACCATCCCTCAA | 68 bp |
|  | R | CAAACAAAGTAAAGCCCAAAGA |  |
|  | Probe | (HEX)-TGCAGTTCTGTCCCTGTAGGCCA-(BHQ1) |  |
| Bm1_49345  (Bm12920)  Histone H3 | F | AAATTGCAACGAATGTGTCC | 140 bp |
|  | R | ACGTTTCGCATGAATAGCAG |  |
|  | Probe | (HEX)-TTCCGCAGCTTCCTGTAGAGCTGA-(BHQ1) |  |
