# Supplementary material for: Effects of diethylcarbamazine and ivermectin treatment on Brugia malayi gene expression in infected gerbils (Meriones unguiculatus)
Source: Parasitol Open. Author manuscript; Available in PMC 2021 Mar 26. (PMC7994942; doi:10.1017/pao.2019.1)
Supplement: Supplementary Table 2 [file NIHMS1519550-supplement-Supplementary_Table_2.docx]

*Supplementary Table 2*. Transcriptomic and Genomic Coverage

| 819,917,610 Adult paired reads | 296,909,192 Mf paired reads |
| --- | --- |
| Adult Overall mapping: 94.4% | Mf Overall mapping: 82.9% |
| Adult Concordant mapping: 91.8% | Mf Concordant mapping: 80.5% |
| Adult DEG: 119 | Mf DEG: 84 |
