# Supplementary material for: Effects of diethylcarbamazine and ivermectin treatment on Brugia malayi gene expression in infected gerbils (Meriones unguiculatus)
Source: Parasitol Open. Author manuscript; Available in PMC 2021 Mar 26. (PMC7994942; doi:10.1017/pao.2019.1)
Supplement: Supplementary Table 3 [file NIHMS1519550-supplement-Supplementary_Table_3.pdf]

Supplementary Table 3. Lists of DEG, together with their O. volvulus and C. elegans orthologs and the extent of change in their expression level after drug treatment.

ALB females 7 days

Sequence	WormBase Gene ID	log2 fold change	FDR	*C. elegans* ortholog	*O. volvulus* ortholog
Bm7847	WBGene00228108	-0.125207884	0.02836575		
Bm10099	WBGene00230360	-0.126530684	0.035017781		OVOC10734
Bm2841	WBGene00223102	-0.162015101	0.02836575	*oac-9*	*Ovo-oac-9*
Bm10602	WBGene00230863	-0.179499492	0.003146962		OVOC6357, OVOC6365
Bm17065	WBGene00255705	-0.191656485	0.000123418		
Bm8178	WBGene00228439	-0.313084383	0.02836575	K05F1.9, ZK354.7, ZK688.[illegible]	OVOC9922

IVM Mf 24hrs

Sequence	WormBase Gene ID	log2 fold change	FDR	*C. elegans* ortholog	*O. volvulus* ortholog
Bm3229	WBGene00223490	0.603123218	0.0081356	*msh-5*	*Ovo-msh-5*
Bm4715	WBGene00224976	0.602473275	0.007382736	*cbn-1*	*Ovo-cbn-1*
Bm8106	WBGene00228367	0.600167168	0.0081356	*mig-6*	
Bm4155	WBGene00224416	0.587393141	0.005066018	*cey-2* , *cey-3* , *cey-4*	OVOC9790
Bm2740	WBGene00223001	0.572797758	0.005575019		*Ovo-aff-1*
Bm6109	WBGene00226370	0.564215969	0.014575934	*mlt-11*	OVOC7267
Bm6642	WBGene00226903	0.562107113	0.012486949	*men-1*	OVOC10389
Bm2857	WBGene00223118	0.556934404	0.014809739	C50B8.6	OVOC9598
Bm16945	WBGene00255599	0.545797965	0.000761748		OVOC1347, OVOC3455, OVOC7593, OVOC7614, OVOC11994, OVOC12014
Bm9375	WBGene00229636	0.540266945	0.008745337		OVOC7185
Bm17728	WBGene00268870	0.534389578	0.000483777		
Bm1598	WBGene00221859	0.531009082	0.005575019		OVOC6427
Bm5637	WBGene00225898	0.527471655	0.0333042	*tbx-43* , *tbx-42* , *tbx-40*	OVOC9248
Bm3156	WBGene00223417	0.50831588	0.003876134	*wht-4*	*Ovo-wht-4*
Bm4913	WBGene00225174	0.502923605	0.011624049	*tyr-2*	OVOC7558
Bm1529	WBGene00221790	0.492729715	0.003876134		
Bm4322	WBGene00224583	0.490784952	0.011624049	F26E4.5	OVOC8230
Bm3166	WBGene00223427	0.487724281	0.037483462	F07C3.3, M03F8.5, Y38A10A.2	OVOC2558
Bm9105	WBGene00229366	0.468974295	0.012486949		
Bm9170	WBGene00229431	0.464739581	0.0081356		
Bm9046	WBGene00229307	0.45149796	0.047249541	*him-3* , *htp-1* , *htp-2*	OVOC1489
Bm2775	WBGene00223036	0.448763519	0.024925705	*ptr-4*	*Ovo-ptr-4*
Bm7475	WBGene00227736	0.436376564	0.011624049	*gcy-9*	*Ovo-gcy-9*
Bm11511	WBGene00231772	0.435144708	0.006520964	*kel-10* and F47D12.7	OVOC5827
Bm7031	WBGene00227292	0.43501553	0.024925705	F25H5.7, T28F4.3, F20H11.4, F55F8.7	OVOC12055
Bm11108	WBGene00231369	0.432340305	0.003876134		OVOC9442
Bm4339	WBGene00224600	0.431582363	0.007939778	F01D5.7 and F01D5.8	OVOC6406, OVOC12639
Bm17409	WBGene00268552	0.430281252	0.011597713		OVOC1347, OVOC1433, OVOC7593, OVOC7614, OVOC9670, OVOC11994, OVOC12014
Bm17720	WBGene00268862	0.42953666	0.0081356		OVOC1347, OVOC1433, OVOC7593, OVOC7614, OVOC9670, OVOC11994, OVOC12014
Bm1395	WBGene00221656	0.428689353	0.015629205		OVOC8114
Bm7583	WBGene00227844	0.425943636	0.011624049	*tba-8*	*Ovo-tba-8*
Bm18179	WBGene00269319	0.419436527	0.040177072	F29G6.1 and ZK813.6	
Bm9103	WBGene00229364	0.41939594	0.050342906		OVOC9032
Bm17184	WBGene00268327	0.418825781	0.01515549		OVOC1347, OVOC1433, OVOC7593, OVOC7614, OVOC9670, OVOC11994, OVOC12014
Bm2682	WBGene00222943	0.416749139	0.012486949	*cpb-1*	*Ovo-cpb-1*
Bm17645	WBGene00268787	0.41189729	0.005575019		
Bm2098	WBGene00222359	0.405903908	0.052106221	*lips-7*	*Ovo-lips-7*
Bm11609	WBGene00231870	0.405627142	0.021797719	*tba-9*	OVOC12330
Bm11532	WBGene00231793	0.399641338	0.022504593	*kri-1*	*Ovo-kri-1*
Bm10033	WBGene00230294	0.399412677	0.008247734		
Bm12985	WBGene00233246	0.399231435	0.03407774	*aex-5*	*Ovo-aex-5*
Bm9171	WBGene00229432	0.393491865	0.026781924		OVOC4607
Bm1548	WBGene00221809	0.388841059	0.033736216		OVOC12306
Bm12975	WBGene00233236	0.387130245	0.015629205	*cpl-1*	OVOC1748
Bm18064	WBGene00269204	0.386939162	0.02991671	C02B8.12	OVOC2003

Bm10331	WBGene00230592	0.38065177	0.047405999		OVOC10883
Bm9487	WBGene00229748	0.369060018	0.021744866	F53E4.1	
Bm17721	WBGene00268863	0.366801038	0.025992486	*glb-22*	
Bm10251	WBGene00230512	0.364146975	0.025992486		OVOC11599 and OVOC12781
Bm1106	WBGene00221367	0.364124528	0.050967245		
Bm1551	WBGene00221812	0.360641975	0.050342906		
Bm8206	WBGene00228467	0.357273968	0.011624049		OVOC12590
Bm17834	WBGene00268976	0.351452779	0.050967245		OVOC12004
Bm14333	WBGene00234594	0.345511345	0.047249541	*cpg-1* , *cbd-1* , *cpg-2* , C39D10.7, R02F2.4	OVOC3490
Bm9585	WBGene00229846	0.34365545	0.04340199	*inx-14*	*Ovo-inx-14*
Bm8327	WBGene00228588	0.341849724	0.047249541		OVOC12449
Bm8897	WBGene00229158	0.331701301	0.040177072		OVOC3729
Bm1560	WBGene00221821	0.328726555	0.037546831	*lpd-3*	
Bm7979	WBGene00228240	0.31750008	0.014809739		
Bm4929	WBGene00225190	0.304624789	0.050967245	*fut-1*	*Ovo-fut-1*
Bm795	WBGene00221056	0.30432317	0.050342906	T19C3.5	
Bm7560	WBGene00227821	-0.355766822	0.050967245	ZK829.7	OVOC4076
Bm15604	WBGene00235501	-0.415553399	0.050342906		
Bm7804	WBGene00228065	-0.425086888	0.052106221		OVOC9587
Bm4931	WBGene00225192	-0.434899547	0.047249541	*let-19*	*Ovo-let-19*
Bm4815	WBGene00225076	-0.494911422	0.005575019	*ogt-1*	*Ovo-ogt-1*
Bm17679	WBGene00268821	-0.502588189	0.047249541		
Bm3628	WBGene00223889	-0.511491901	0.0333042	*nhl-2*	*Ovo-nhl-2*
Bm9130	WBGene00229391	-0.5243214	0.024925705	*ccch-1*	*Ovo-ccch-1*
Bm8701	WBGene00228962	-0.566703114	0.011624049		OVOC11981
Bm9830	WBGene00230091	-0.585257744	0.003876134		OVOC10471
Bm4360	WBGene00224621	-0.585940801	0.00767797	*dnj-13*	OVOC11163
Bm11366	WBGene00231627	-0.600457693	0.005575019	*anc-1*	*Ovo-anc-1*

IVM males 24hrs

Sequence	WormBase Gene ID	log2 fold change	FDR	*C. elegans* ortholog	*O. volvulus* ortholog
Bm3485	WBGene00223746	-0.323118946	0.025504201	F21C10.7	OVOC6983
Bm5769	WBGene00226030	-0.354783159	0.038154344	*pis-1*	*Ovo-pis-1*
Bm2799	WBGene00223060	-0.378407472	0.049368954	*pqn-22*	*Ovo-pqn-22*
Bm9628	WBGene00229889	-0.400305222	0.049368954		OVOC4735
Bm12835	WBGene00233096	-0.40942616	0.038154344	csp-2, D2096.11, M116.5	*Ovo-csp-2*
Bm7648	WBGene00227909	-0.418654974	0.038154344	*lec-5*	*Ovo-lec-5*
Bm10580	WBGene00230841	-0.430384697	0.025504201		OVOC6940
Bm3817	WBGene00224078	-0.454452749	0.038154344	F35A5.1	OVOC2579
Bm5950	WBGene00226211	-0.457973484	0.038154344	T23E7.2	OVOC4530
Bm6544	WBGene00226805	-0.482889752	0.025504201	Y43F8B.1	OVOC6340
Bm7502	WBGene00227763	-0.502328748	0.025504201	*unc-22*	*Ovo-unc-22*
Bm11186	WBGene00231447	-0.51020492	0.025504201	*myo-3*	
Bm4116	WBGene00224377	-0.530126274	0.013822439	*myo-1, myo-2* and *unc-54*	*Ovo-unc-54*
Bm6220	WBGene00226481	-0.613328636	0.009739043	*ttn-1*	OVOC7180

IVM males 7 days

Sequence	WormBase Gene ID	log2 fold change	FDR	*C. elegans* ortholog	*O. volvulus* ortholog
Bm3610	WBGene00223871	0.669709305	0.006886427	T14B4.9, R07C3.3, H41C03.3, ZK1225.2, Y51H4A.25, T28F3.9, T27F6.1, T09E11.9, T09E11.6, R07B7.6, F30A10.4 and F26D2.3	OVOC2556
Bm7894	WBGene00228155	0.664706797	0.000679705		OVOC7570
Bm876	WBGene00221137	0.621393902	0.047730723		OVOC7056
Bm9729	WBGene00229990	0.617090083	0.022757308		OVOC8182
Bm9021	WBGene00229282	0.595334071	0.011145411	*col-8, col-19, col-38, col-41, col-49, col-90, col-106, col-140, col-175, col-184,* and *dpy-5*	
Bm2510	WBGene00222771	0.560936509	0.000373798	C32D5.8, *trx-5* , T20D4.7 and T28A11.13	OVOC9847
Bm17065	WBGene00255705	0.560632424	0.002578222		
Bm8043	WBGene00228304	0.547967126	0.034109131		OVOC3168
Bm9853	WBGene00230114	0.525906045	0.028856033		
Bm6240	WBGene00226501	0.431998862	0.035293538	*mlt-8*	*Ovo-mlt-8*
Bm2632	WBGene00222893	0.36277445	0.002578222	*mlc-1* and *mlc-2*	
Bm2216	WBGene00222477	0.32475724	0.00484332	C15C7.5	OVOC3684
Bm4489	WBGene00224750	-0.344332628	0.022697033	F56F3.4	OVOC2534
Bm12054	WBGene00232315	-0.382899813	0.034658288	*che-12*	*Ovo-che-12*
Bm12109	WBGene00232370	-0.581766917	0.034109131		OVOC9300

IVM females 7 days

Sequence	WormBase Gene ID	log2 fold change	FDR	*C. elegans* ortholog	*O. volvulus* ortholog
Bm5144	WBGene00225405	0.405082259	0.011685256	*hil-1*	*Ovo-hil-1*
Bm4699	WBGene00224960	0.362208076	0.01227879	H42K12.3	OVOC5777
Bm6215	WBGene00226476	0.343166521	0.01227879	*lit-1*	*Ovo-lit-1*
Bm6122	WBGene00226383	0.34087873	0.014637872		
Bm17689	WBGene00268831	0.33330738	0.045930921	*nars-1*	OVOC6597
Bm6123	WBGene00226384	0.315713822	0.048008363	*hmr-1* and Y52B11A.11	OVOC253
Bm6781	WBGene00227042	0.315010689	0.030044579	*smg-9*	*Ovo-smg-9*
Bm3958	WBGene00224219	0.314397144	0.042813154	*sox-2*	OVOC3659
Bm6109	WBGene00226370	0.300095453	0.049013686	*mlt-11*	OVOC7267
Bm12270	WBGene00232531	0.29418257	0.030044579	*spv-1*	OVOC11050
Bm1811	WBGene00222072	0.290289389	0.044857682	*lat-1*	*Ovo-lat-1*
Bm5103	WBGene00225364	0.286451395	0.046094181	*hlh-2*	OVOC120, OVOC12718
Bm8236	WBGene00228497	-0.100693715	0.045930921		OVOC2846
Bm8780	WBGene00229041	-0.105971798	0.025079902		
Bm8137	WBGene00228398	-0.11495815	0.040170044		
Bm7847	WBGene00228108	-0.121703444	0.022690208		
Bm1094	WBGene00221355	-0.126435859	0.030649281		
Bm6160	WBGene00226421	-0.130017793	0.049013686	T16A9.5, Y37D8A.5, Y69E1A.1, K06A5.2, W03D8.9	
Bm10099	WBGene00230360	-0.133041222	0.01227879		OVOC10734
Bm9015	WBGene00229276	-0.141346968	0.022690208		
Bm2841	WBGene00223102	-0.162374427	0.014637872	*oac-9*	*Ovo-oac-9*
Bm10602	WBGene00230863	-0.180931318	0.00240351		OVOC6357, OVOC6365
Bm17065	WBGene00255705	-0.182830196	0.000480593		
Bm12369	WBGene00232630	-0.25435489	0.045930921		OVOC9443
Bm6402	WBGene00226663	-0.318671794	0.022690208	Y37E11AM.2	

IVM Mf 7 days

Sequence	WormBase Gene ID	log2 fold change	FDR	*C. elegans* ortholog	*O. volvulus* ortholog
Bm41	WBGene00220302	-0.473735508	0.005641028		

DEC males 24 hrs

Sequence	WormBase Gene ID	log2 fold change	FDR	*C. elegans* ortholog	*O. volvulus* ortholog
Bm8476	WBGene00228737	0.651980266	0.000582997	*cdh-1*	*Ovo-cdh-3*
Bm3892	WBGene00224153	-0.289273911	0.019001004	F37C12.1	OVOC8251
Bm3645	WBGene00223906	-0.372119571	0.000910611	F27C1.6	OVOC13453
Bm3760	WBGene00224021	-0.376020143	0.019001004	*fipp-1*	*Ovo-fipp-1*
Bm13809	WBGene00234070	-0.38830739	0.010407687	H06I04.3	OVOC8075

DEC females 7 days

Sequence	WormBase Gene ID	log2 fold change	FDR	*C. elegans* ortholog	*O. volvulus* ortholog
Bm5103	WBGene00225364	0.382436568	0.000820917	*hlh-2*	OVOC120, OVOC12718
Bm6122	WBGene00226383	0.349976384	0.003770296		
Bm3958	WBGene00224219	0.343503359	0.003770296	*sox-2*	OVOC3659
Bm12262	WBGene00232523	0.342192779	0.005266159	*sec-16* and F13B9.1	OVOC1844
Bm6960	WBGene00227221	0.334522654	0.007930108	*tfg-1* and Y71A12B.10	OVOC295
Bm4234	WBGene00224495	0.326985825	0.008658882	*efl-3*	Ovo-efl-3
Bm6123	WBGene00226384	0.32540846	0.011246577	*hmr-1* and Y52B11A.11	OVOC253
Bm6084	WBGene00226345	0.322930881	0.011246577	*npa-1*	*Ovo-npa-1*
Bm3276	WBGene00223537	0.317053132	0.01325891	*mig-10*	OVOC9227
Bm17512	WBGene00268655	0.310294655	0.013837549		OVOC3551
Bm5144	WBGene00225405	0.3093214	0.016991278	*hil-1*	*Ovo-hil-1*
Bm3368	WBGene00223629	0.296813439	0.016991278	F14E5.2	OVOC10996
Bm4063	WBGene00224324	0.289798532	0.016991278	*patr-1*	*Ovo-patr-1*
Bm8119	WBGene00228380	0.285264222	0.016991278		OVOC8194
Bm9676	WBGene00229937	0.281537644	0.019677731	*cdt-1*	*Ovo-cdt-1*
Bm3500	WBGene00223761	0.28041483	0.02081835	*ptc-1* and *ptc-2*	OVOC11071
Bm9098	WBGene00229359	0.275792615	0.025048585	*gei-8*	*Ovo-gei-8*
Bm6215	WBGene00226476	0.27429962	0.025048585	*lit-1*	*Ovo-lit-1*
Bm5327	WBGene00225588	0.273253383	0.025048585	*cbp-1* , *cbp-2* , F40F12.7	OVOC7710
Bm7304	WBGene00227565	0.270168748	0.025048585	*swsn-1*	*Ovo-swsn-1*
Bm1811	WBGene00222072	0.267255747	0.025048585	*lat-1*	*Ovo-lat-1*
Bm5288	WBGene00225549	0.265789355	0.025048585	*npp-21*	OVOC10101
Bm1770	WBGene00222031	0.260327005	0.025048585	*unc-44*	*Ovo-unc-44*
Bm12943	WBGene00233204	0.259140955	0.025048585		OVOC729
Bm3046	WBGene00223307	0.251566803	0.025988087	*atx-2*	*Ovo-atx-2*
Bm10372	WBGene00230633	0.249766833	0.028716172		*Ovo-din-1*
Bm6167	WBGene00226428	0.247167353	0.028716172	*afd-1*	*Ovo-afd-1*
Bm5397	WBGene00225658	0.246917715	0.031444362	*sma-1*	*Ovo-sma-1*
Bm4925	WBGene00225186	0.246435076	0.034565441	*ctf-18*	OVOC4191
Bm405	WBGene00220666	0.244045099	0.034565441	*npp-11*	OVOC2089
Bm3036	WBGene00223297	0.240578954	0.034565441	*utx-1* , *jmjd-3.1* , *jmjd-3.2*	*Ovo-utx-1*
Bm11336	WBGene00231597	0.237661771	0.034741174	*evl-14*	*Ovo-evl-14*
Bm4682	WBGene00224943	0.234435341	0.035142865	*sacy-1*	*Ovo-sacy-1*

Bm12511	WBGene00232772	0.233976236	0.035673633	K07H8.10	OVOC3812
Bm5544	WBGene00225805	0.233631383	0.035697505	*act-5*	OVOC8134
Bm5390	WBGene00225651	0.230124536	0.035697505	*hmp-1*	*Ovo-hmp-1*
Bm14237	WBGene00234498	0.22900569	0.035697505		OVOC122
Bm11206	WBGene00231467	0.225509143	0.035697505		OVOC11021
Bm3633	WBGene00223894	0.221432761	0.035697505	*chd-3* and *let-418*	OVOC9926
Bm7465	WBGene00227726	0.221275512	0.035697505	*sax-3*	OVOC5391
Bm4482	WBGene00224743	0.220573829	0.035697505	*vig-1*	*Ovo-vig-1*
Bm4917	WBGene00225178	0.219890489	0.037911503	*spt-5*	*Ovo-mfap-1*
Bm10506	WBGene00230767	0.216328005	0.037911503		OVOC7376
Bm2081	WBGene00222342	0.213617541	0.037911503	*vgln-1*	OVOC9684
Bm12925	WBGene00233186	0.2098293	0.037911503		OVOC786
Bm2204	WBGene00222465	0.207909225	0.037911503	*sfxn-2*	*Ovo-sfxn-2*
Bm2358	WBGene00222619	0.205378839	0.038553083	*dao-5*	*Ovo-dao-5*
Bm12579	WBGene00232840	0.202292278	0.041402625	*emb-5*	*Ovo-emb-5*
Bm5543	WBGene00225804	0.192858537	0.047998464	*epn-1*	*Ovo-epn-1*
Bm1868	WBGene00222129	-0.219640958	0.047998464	C01B10.11	OVOC4533
Bm7924	WBGene00228185	-0.236465839	0.047998464		OVOC7728
Bm5335	WBGene00225596	-0.256650575	0.047998464	*lpd-8*	*Ovo-lpd-8*
Bm17568	WBGene00268711	-0.279130863	0.047998464	*lec-3*	*Ovo-lec-3*
Bm5648	WBGene00225909	-0.39386824	0.000304444		OVOC8243

DEC Mf 7 days

Sequence	WormBase Gene ID	log2 fold change	FDR	*C. elegans* ortholog	*O. volvulus* ortholog
Bm1750	WBGene00222011	0.399043298	0.048675157	*hlh-1*	*Ovo-hlh-1*
Bm17998	WBGene00269140	-0.35889784	0.048675157		
Bm4711	WBGene00224972	-0.368759952	0.048675157		*Ovo-ttr-51*
Bm5423	WBGene00225684	-0.378713626	0.013820385	*larp-1*	*Ovo-larp-1*
Bm9702	WBGene00229963	-0.403417906	0.013197683		
Bm1947	WBGene00222208	-0.432182624	0.005127346	*pac-1*	*Ovo-pac-1*
Bm10347	WBGene00230608	-0.468563334	0.013197683		OVOC11012
Bm3354	WBGene00223615	-0.469359323	0.001009721	*bcl-11*	OVOC7199
Bm4783	WBGene00225044	-0.538725623	0.000725039	*fkh-9*	*Ovo-fkh-9*
Bm41	WBGene00220302	-1.535093715	2.74E-55		
