# Supplementary material for: Effects of diethylcarbamazine and ivermectin treatment on Brugia malayi gene expression in infected gerbils (Meriones unguiculatus)
Source: Parasitol Open. Author manuscript; Available in PMC 2021 Mar 26. (PMC7994942; doi:10.1017/pao.2019.1)
Supplement: Supplementary Table 4 [file NIHMS1519550-supplement-Supplementary_Table_4.docx]

**Supplementary Table 4**. Comparison of fold expression changes given by DESeq2 vs qPCR

| qPCR target gene | *C. elegans* ortholog | Treatment leading to change in expression | 2^DESeqlog2^ | 2^-ΔΔCt^ qPCR |
| --- | --- | --- | --- | --- |
| Bm4155 | *cey-2*;*cey-3* | ↑ IVM Mf 24 hours | 1.5025 | 1.27 |
| Bm4360 | *dnj-13* | ↓ IVM Mf 24 hours | 0.666 | 0.175 |
| Bm1750 | *hlh-1* | ↑ DEC Mf 7 days | 1.3186 | 4.25 |
| Bm4783 | *fkh-9* | ↓ DEC Mf 7 days | 0.688 | 0.545 |
| Bm7847 | None | ↓ IVM ♀ 7 days | 0.919 | 0.00379 |
| Bm7847 | None | ↓ ALB ♀ 7 days | 0.916 | 0.0024 |
| Bm5185 | None | ↓ IVM ♀ 7 days | 0.913 | 0.2798 |
| Bm6220 | *ttn-1* | ↓ IVM ♂ 24 hours | 0.655 | 0.593 |
| Bm3390 | *ubql-1* | ↑ DEC ♀ 24 hours | 1.6 | 1.901 |
| Bm4605 | *col-119* | ↑ IVM ♂ 7 days | 1.69 | 2.69 |
