# Supplementary material for: Effects of diethylcarbamazine and ivermectin treatment on Brugia malayi gene expression in infected gerbils (Meriones unguiculatus)
Source: Parasitol Open. Author manuscript; Available in PMC 2021 Mar 26. (PMC7994942; doi:10.1017/pao.2019.1)
Supplement: Supplementary Table 5 [file NIHMS1519550-supplement-Supplementary_Table_5.docx]

**Supplementary Table 5.** Differentially expressed genes following ivermectin treatment of *B. malayi* whose *C. elegans* orthologs are differentially expressed in response to stress.

| **Gene** | **Function** | **Stage where differentially expressed** |
| --- | --- | --- |
| Hil-1 | H1 histone family member | Most upregulated in 7-day females. |
| H42K12.3 |  | 2nd most upregulated in 7-day females |
| Smg-9 | Nonsense mediated mRNA decay factor | Upregulated in 7-day females. |
| T16A9.5 |  | Downregulated in 7-day females. |
| Y43F8B.1 |  | Downregulated in 24-hr males |
| T23E7.2 |  | Downregulated in 24-hr males |
| C02B8.12 |  | Up-regulated in 24hr Mf |
| Dnj-13 | DnaJ domain (heat-shock protein) | 2nd most downregulated in 24hr Mf |
| F21C10.7 |  | Downregulated in 24hr males |
| C50B8.6 |  | Upregulated in 24 hr Mf |
| Wht-4 | ABCG protein | Upregulated in 24hr Mf |
| Kel-10 | Kelch repeat and BTB domain | Upregulated in 24hr Mf |
| F01D5.7 | Abhydrolase domain containing | Upregulated in 24hr Mf |
| Trx-5 | Nucleoredoxin-like | Upregulated in 7day males |
